# The Efficacy of Digital Cognitive–Behavioral Interventions in Supporting the Psychological Adjustment and Sleep Quality of Pregnant Women with Sub-Clinical Symptoms: A Systematic Review and Meta-Analysis

**DOI:** 10.3390/ijerph19159549

**Published:** 2022-08-03

**Authors:** Elisa Mancinelli, Giulia Bassi, Silvia Gabrielli, Silvia Salcuni

**Affiliations:** 1 Department of Developmental and Socialization Psychology, University of Padova, Via Venezia 8, 35121 Padova, Italy; giulia.bassi@unipd.it (G.B.); silvia.salcuni@unipd.it (S.S.); 2 Digital Health Lab, Centre for Digital Health and Wellbeing, Fondazione Bruno Kessler, Via Sommarive 18, 38123 Trento, Italy; sgabrielli@fbk.eu

**Keywords:** treatment efficacy, pregnancy, sub-clinical symptoms, psychological adjustment, sleep quality, digital interventions, cognitive–behavioral therapy (CBT), third-generation CBT, meta-analysis

## Abstract

The present meta-analysis investigated the overall and differential efficacy of digital cognitive–behavioral therapies (CBTs) vs. third-generation CBTs deployed to pregnant women in reducing sub-clinical depression, anxiety, and stress symptoms while fostering sleep quality and quality of life. A PRISMA-guided systematic search was used, including randomized controlled trials (RCTs) evaluating the above-mentioned interventions. Data were pooled using either the mean difference (MD) or standardized MD (SMD). Sub-group analyses were carried out when appropriate. The primary outcomes were depression, anxiety, and stress symptoms, as well as sleep quality and quality of life. The interventions’ acceptability was evaluated through the odds ratio (OR) of drop-out rates. Seven RCTs were included, comprising 1873 pregnant women. The results showed the interventions’ efficacy in terms of reducing depression symptoms (SMD = −0.36, CI = 0.61, −0.11, k = 9) at the endpoint, although it was not maintained at follow-up during the postpartum period. The interventions’ efficacy in terms of reducing anxiety symptoms (SMD = 1.96, CI = −2.72, −1.21, k = 3) at the endpoint was also significant, while having no effect on sleep quality. The interventions were well accepted (OR = 1.68; 95% CI = 0.84, 3.35; k = 7). Although no sound conclusions can be drawn concerning the joint or differential efficacy of the considered interventions, this study was useful in highlighting the need to develop evidence-based digital prevention programs for pregnant women with sub-clinical symptoms.

## 1. Introduction

Pregnancy is a complex period, whereby the transition to motherhood is lived as a life-changing event, so much so that it entails an “identity reorganization” requiring women to transform and then restore their self-identity [1]. During this period many physiological, social, and psychological changes occur, and the ways women adapt to these changes can have an influence on their quality of life and well-being and on the possibility of developing emotional difficulties and mental health problems [2]. Getting pregnant and becoming a mother, as periods of vulnerability, are often constellated by increasing worries and fear of not being able to cope with all the changes and the child on the way [3]. Pregnancy is also associated with increased hormonal fluctuations and continuous bodily changes that altogether contribute to the emotional lability that is commonly experienced during pregnancy [3]. Indeed, perinatal women often experience euphoria, irritability, and depression symptoms both during pregnancy and later during the postpartum period [4,5]. In this regard, it is noteworthy that the stressfulness inherent to pregnancy is linked with an hyperactivity of the hypothalamic–pituitary–adrenal axis (HPA) [6], which is fundamental for the regulation of the stress response. The hyperactivity of the HPA, which is associated with heightened psychological stress, is deemed as one of the etiological factors leading to increased depression symptoms [7,8], which are highly comorbid with anxiety symptoms [9,10]. Indeed, depression, anxiety, and stress symptoms, beyond being highly interrelated [11,12,13,14], are all quite prevalent during pregnancy (depression: 15–65% [15]; anxiety: 18–24% [16]; stress: low–moderate 78% [17]), as well as during the postpartum period (depression: around 17% [18]; anxiety: around 15% [16]; stress: 20–40% [19]). The increased stress response and psychosocial symptoms during pregnancy jointly contribute to hindering women’s perinatal psychological adjustment and significantly increase the risk of developing postpartum depression [20,21]. Furthermore, depression, anxiety, and stress symptoms are also interrelated with reduced sleep quality [22,23,24,25], which is also common during pregnancy, as shown by over 50% of pregnant women [26,27]. These symptoms and difficulties contribute to reducing the overall quality of life of pregnant women [25,28], with implications for their psychosocial functioning and later for that of their children [29,30,31,32,33,34].

The above factors altogether highlight the motives, whereby prevention and intervention programs aimed at fostering pregnant women’s psychosocial adjustment might be particularly valuable in reducing the chances of developing clinically relevant psychological symptoms while favoring their quality of life and well-being. It is worth noting that interventions deployed through digital means might be particularly valuable in providing brief, timely, and easily accessible support, which would ultimately allow scalability [35,36], thereby reaching an increasing number of women with reduced timeframes and fewer costs. In this regard, cognitive–behavioral therapies (CBTs) and third-generation CBTs, which are based on specific protocols and include practical activities, might be particularly fitted to be implemented via digital solutions. The core difference between traditional CBTs and third-generation CBTs is that the former rely on the principles of classical and operant conditioning, and through those acts on the dysfunctional thought processes that guide maladaptive situational appraisal. In contrast, third-generation CBTs are greatly focused on enhancing experiential and contextual awareness by favoring more adaptive coping strategies [37,38]. Indeed, third-wave CBTs give priority to the holistic enhancement of psychological and behavioral processes associated with health and well-being [37]. Among some of the main third-generation CBTs are mindfulness-based cognitive therapy (MBCT), mindfulness-based stress reduction (MBSR), acceptance and commitment therapy (ACT), and dialectical Behavioral therapy (DBT) [37,38].

Previous reviews and meta-analyses have already highlighted the efficacy of in-person CBTs deployed during the perinatal period in reducing depression, anxiety, and stress symptoms [39,40]. However, the efficacy of digital CBTs seems only supported for a reduction in depression symptoms [41,42], since to date no review has been conducted to assess their efficacy in reducing anxiety or stress symptoms. On a similar note, the efficacy of digital CBTs in improving insomnia symptoms was reported for adults at large [43], yet there are no available reviews or meta-analyses focused on perinatal sleep issues.

Distinguishing between CBTs and third-generation CBTs, it is worth noting that as just mentioned, while the former seems effective in supporting perinatal women’s mood disturbances, the results are unclear for third-generation CBTs [44,45,46]. Indeed, the small number of reviews and meta-analyses carried out in this regard, which were focused on in-person mindfulness-based interventions (MBIs), showed that these interventions were not superior to the control conditions in reducing depression, anxiety, or stress symptoms [44,45]. Furthermore, in a recent meta-analysis, albeit where the authors reported a small effect on depression symptoms during pregnancy [46], the efficacy of MBIs on stress symptoms was unclear and that on anxiety symptoms could not be assessed due to a lack of studies. Regarding digital interventions, to our current knowledge no review study has been conducted to investigate the efficacy of digital third-generation CBTs deployed during the perinatal period.

A further evaluation of the available literature highlighted that studies investigating the efficacy of CBTs and third-generation CBTs have greatly focused on women in the postpartum period [47,48] or on the broader perinatal period (i.e., both ante- and postnatal) [49]. Moreover, these studies were based either on specific intervention protocols (e.g., traditional CBTs) [41,42] or conversely included psychological interventions at large [50,51], despite their specific theoretical background. In addition, among the studies that placed attention on pregnancy, the involved women presented either clinically relevant psychological symptoms or mental disorders, such as major depression [52,53], or were instead recruited from the general population regardless of their symptomatology level [41,42,54]. The only available study that solely included women with sub-clinical symptoms and no prior mental disorder considered the broader perinatal period and investigated the efficacy of in-person MBIs [46]. Therefore, being mindful of the above and with a specific interest in prevention, the present meta-analysis is aimed at providing further and more specific information useful to the development of digital interventions and applications within a cognitive–behavioral approach, which can favor and support the well-being and mental health of pregnant women from the general population showing sub-clinical symptoms. More specifically, to our knowledge this is the first study that intends to evaluate the overall and differential efficacy of digital CBTs vs. third-generation CBTs in reducing sub-clinical depression, anxiety, and stress symptoms while fostering sleep quality and the overall quality of life of pregnant women. The specificities of the different interventions and the women’s characteristics (e.g., ethnicity, gestational week, etc.) will also be taken into account to try and identify potential moderators of the interventions’ efficacy.

This is altogether expected to be valuable for the development and implementation of appropriate prevention and intervention programs tailored to the peculiar needs and characteristics of the mentioned population. Indeed, digital interventions originating from a cognitive–behavioral approach have the potential to support the psychological adjustment of pregnant women and to prevent the development of clinically relevant symptoms by providing transversal strategies useful to deal with everyday stressors, thereby supporting their psychological adjustment. Digital solutions can allow pregnant women to save time and costs, since the interventions are deployed online, making them much more easily accessible and also reducing help-seeking barriers [55]. This resonates with the stepped care model, which aims at fostering the spread of mental health programs by supporting the coordination between primary and secondary mental health services [56].

## 2. Materials and Methods

The protocol for this meta-analysis was accepted and registered on the PROSPERO website in December 2021 (Registration Number: CRD42021289436).

The present meta-analysis was carried out in accordance with the Preferred Reporting Items for Systematic Reviews and Meta-Analyses (PRISMA; see the Supplementary Materials Table S1) guidelines [57] and the Cochrane Handbook for Systematic Reviews recommendations [58].

### 2.1. Eligibility Criteria

Studies that fulfilled the following criteria were included in the present study: (a) women experiencing physiological pregnancy; (b) pregnant women aged ≥ 18; (c) being a randomized controlled trial (RCT); (d) RCTs investigating the efficacy of digital CBTs and third-generation CBTs; (e) RCTs comparing digital CBTs (i.e., third-generation CBTs and CBTs) with any active or inactive control condition (e.g., no intervention group, treatment as usual (TAU), waiting list). On the other hand, studies that satisfied the following criteria were excluded from the current meta-analysis: (a) women with a past or present history of mental disorders; (b) women with clinically significant psychological symptoms (i.e., over the clinical cut-off of the administered questionnaires); (c) women with an at-risk pregnancy; (d) women presenting medical conditions, either pregnancy-related or not; (e) women experiencing artificially induced pregnancy; (f) conference abstract and thesis.

### 2.2. Primary and Secondary Outcomes

The primary outcomes investigated by the current meta-analysis were depression, anxiety, and stress symptoms, as well as sleep quality and quality of life. The time points related to this meta-analysis were the interventions’ endpoints, considered as the end of the intervention, and follow-ups during the postpartum period (when evaluated). The secondary outcomes were the efficacy of the interventions at follow-up and the acceptability of such interventions assessed through the odds ratio (OR) of drop-out rates.

### 2.3. Search Strategy

Five electronic bibliographic databases (i.e., Web of Science, PubMed, PsycINFO, CINHAL, and Scopus) were systematically screened in December 2021. The search terms applied to the above-mentioned databases were as follows: randomized control trial, cognitive–behavioral intervention, cognitive–behavioral, pregnancy, prenatal, perinatal, depress*, anxiety, stress, quality of life, mindful*, mindfulness interventions, mindfulness-based, mindfulness-based stress reduction, mindfulness-based cognitive therapy, acceptance and commitment therapy, dialectical behavioral therapy. Subsequently, relevant journals and reference lists were manually screened to identify further relevant literature related to the aim of the present meta-analysis. The search was focused only on articles written in English or Italian that were subject to peer-reviewing and that provided the necessary data to perform statistical analyses. Two authors (E.M. and G.B.) independently screened both titles and abstracts in a double-blind fashion. Subsequently, the studies’ eligibility was assessed through full-text screening, which was performed by the same above-mentioned authors in a double-blind fashion. Any conflict that had emerged during the screening process was resolved by consulting a third author (SS) until an agreement was reached.

### 2.4. Data Extraction

Data extraction was independently performed by two authors (E.M. and G.B.), and any disagreement that occurred was resolved by consulting a third author (S.G.). The data extracted from the full-text articles were the following: study DOI, first author name, publication year, population characteristics (i.e., sample size, age, ethnicity, marital or relationships status, socio-economic level, educational level, gestational week, previous pregnancies, functioning at baseline), the cut-off of the measurement tools used for inclusion criteria, study location, type of digital intervention (e.g., web-based, smartphone-based), description of the digital intervention (i.e., means of delivering the intervention, intervention structure, intervention techniques or practices, intervention duration, digital language used), if prior feasibility or pilot studies were conducted, type of control condition (i.e., TAU, active group, waitlist or no treatment), and the type of analyses conducted in the included studies (intention-to-treat (ITT) or per-protocol). Additionally, the outcomes of interest, i.e., the means and standard deviations of anxiety, stress, and depression symptoms, as well as the level of sleep quality and quality of life, were extracted. Lastly, the drop-out rates were recorded separately for each of the RCTs’ trial arms.

### 2.5. Quality Assessment

The quality assessment of the studies was independently carried out by two authors (E.M. and G.B.) in a double-blind fashion using Cochrane’s Risk of Bias tool version 2 (RoB 2.0). Any disagreement that arose between the evaluators was resolved by consulting a third author (S.S.). Each included study was judged based on five domains: the randomization process, deviations from the intended interventions (i.e., effect of assignment to intervention or the effect of adhering to intervention), missing outcome data, measurement of outcome data, and selection of the reported results. Each domain was ranked as “low”, “some concern”, or “high” in terms of the risk of bias. The evaluation of each domain was merged into an overall domain, referred to as the overall assessment, available in the RoB 2.0 to provide a synthesis of the quality assessment of each study in its entirety.

### 2.6. Data Analysis

Comprehensive Meta-Analysis [59] and Review Manager Version 5 (Cochrane, Alberta, Canada) [60] software were used to perform the analyses. The primary and secondary outcomes were meta-analyzed when at least three measurements were available. When the studies assessed an outcome with more than one measurement tool, these were separately included within the analysis. The mean difference (MD; considered when outcomes were assessed with the same measurement tool) or standardized MD (SMD; considered when outcomes were assessed with different measurement tools) were calculated considering the 95% confidence interval (CI). The random-effect model was used when the study heterogeneity exceeded I^2^ > 50%, while the fixed-effect model was used when I^2^ ≤ 50%. The I^2^ index informs on the amount of variance of the interventions’ effect that is imputable to “true” heterogeneity between studies, thereby indicating the studies’ variability. The studies’ acceptability was assessed by calculating the OR of drop-out rates from the intervention and control groups. Sensitivity and subgroup analyses were then performed considering the following moderator variables if available: control group condition (TAU vs. active control), intervention’s theoretical background (CBT vs. third-generation CBT), intervention being based on an intervention full protocol vs. specific techniques or practices, and type of analysis (ITT vs. per-protocol analysis). A meta-regression analysis could not be run, since to be performed data from at least ten studies are needed. Finally, the presence of publication bias was assessed by visually inspecting the funnel plots and by performing Egger’s regression test [61,62]. If publication bias was found, the trim and fill procedure was carried out as well as the fail-safe number [63], thereby verifying whether the findings remain unchanged after accounting for the emerged publication bias.

## 3. Results

### 3.1. Search Results

**Figure 1:**
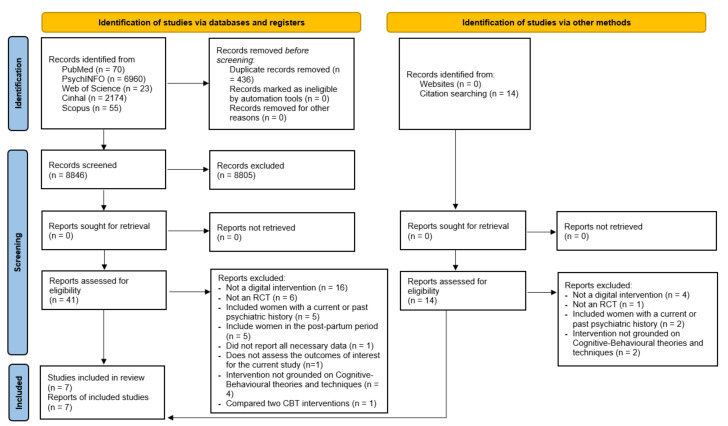
PRISMA 2020 flow diagram, including searches of databases, registers, and other sources [57].

As shown in Figure 1, the initial databases search yielded a total of n = 9282 results. Following the duplicate removal, the titles and abstracts of the resulting n = 8846 studies were screened, of which n = 8805 were excluded. Accordingly, the full texts of n = 41 studies were screened for eligibility. Furthermore, an additional n = 14 studies were retrieved through reference list screening and then screened in line with the inclusion and exclusion criteria. Finally, n = 7 studies were included in the current meta-analysis. The excluded studies with the reasons for exclusion are reported in the Supplementary Materials (Table S2).

### 3.2. Studies Characteristics

Table 1 and Table 2 show, respectively, the samples and intervention characteristics of the included studies (n = 7). Additional information on the samples and study outcomes is reported in the Supplementary Materials (Tables S3 and S4), respectively. 

The studies were published between 2017 and 2021 [64,65,66,67,68,69,70] and comprised N = 1873 pregnant women (M_age_ = 31.03, SD = 1.51). The women’s gestational week at the beginning of the studies was reported in n = 4 studies [67,68,69,70], although Sun and colleagues [69] reported this information in terms of days (intervention group: M_days_ = 97.77, SD = 14.05; control group: M_days_ = 100.85, SD = 15.18). Information on the previous pregnancies was reported by only n = 3 studies [65,67,70]. The samples’ working situation [64,67,69,70] at the time of the study (Supplementary Materials Table S3) and educational level (Table 1) [64,65,67,68] were both reported by n = 4 studies.

Regarding the intervention characteristics (Table 2), n = 4 [65,68,69,70] were based on third-generation CBTs, while n = 3 [64,66,67] were based on traditional CBTs. All third-generation CBTs relied on mindfulness practices, albeit Haga and colleagues’ study [64] was broader, including multiple positive psychology techniques. Furthermore, the interventions were for the most Internet-based, except for n = 2 [69,70], which were smartphone-based. These interventions relied on the Wechat platform, a messaging application used in China. However, while Sun and colleagues [69] deployed their intervention through a custom-built application named “Spirits Healing” with Wechat solely used to send reminders, Yang and colleagues [70] used it as their primary platform to administer the intervention (Table 2).

Of the included RCTs, n = 2 [64,68] were randomized pilot studies and n = 2 [65,67] were RCTs, for which prior pilot studies were conducted. The remaining studies (n = 3) [66,69,70] did not report having previously conducted pilot studies.

Among the outcomes considered by the present meta-analysis (see Supplementary Materials, Table S4), the interventions’ efficacy at the intervention endpoint could be assessed only for depression [64,65,66,67,68,69,70] and anxiety symptoms [67,69,70], as well as sleep quality [66,69], while the interventions’ efficacy in terms of stress symptoms and quality of life could not be assessed, since less than three measurements per time-point were available. Notwithstanding, the 7 studies were quite consistent in the measurement tools used, particularly regarding the assessment of depression symptoms and anxiety symptoms.

### 3.3. Intervention Efficacy and Subgroup Analysis

**Figure 2:**
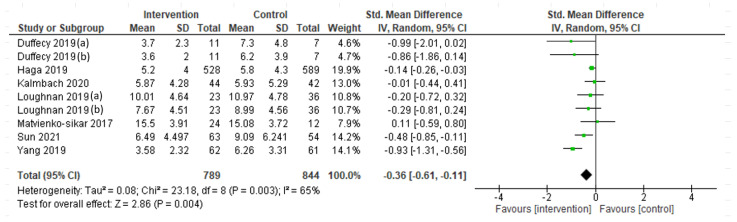
Depressive symptoms at the endpoint: Duffecy 2019 (a, [64]) = PHQ-9; Duffecy 2019 (b, [64]) = HARS; Loughnan 2019 (a, [67]) = EPDS; Loughnan 2019 (b, [67]) = PHQ-9.

**Figure 3:**
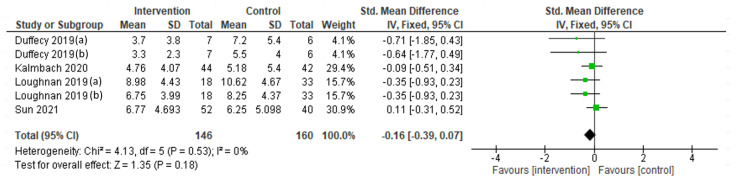
Depression symptoms at the 6-month postpartum follow-up: Duffecy 2019 (a, [64]) = PHQ-9; Duffecy 2019 (b, [64]) = HARS; Loughnan 2019 (a, [67]) = EPDS; Loughnan 2019 (b, [67]) = PHQ-9.

**Figure 4:**
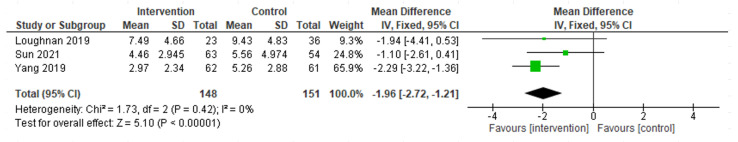
Anxiety symptoms at the endpoint.

**Figure 5:**
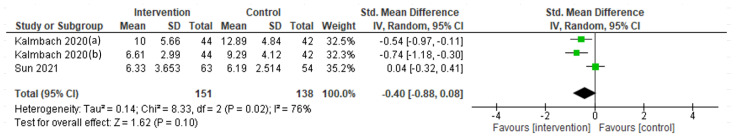
Sleep quality at the endpoint: Kalmbach 2020 (a, [66]) = ISI; Kalmbach 2020 (b, [66]) = PSQI.

**Figure 6:**
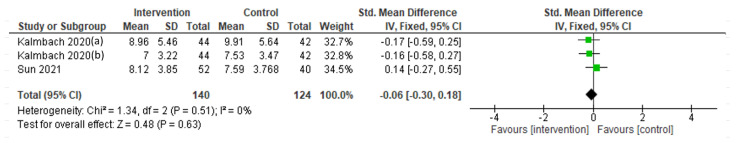
Sleep quality at the 6-month postpartum follow-up: Kalmbach 2020 (a, [66]) = ISI; Kalmbach 2020 (b, [66]) = PSQI.

A random-effect meta-analysis was performed to assess the interventions’ efficacy in terms of depression symptoms at the endpoint (Figure 2), showing a significant reduction (SMD= −0.36; 95% CI = −0.61, −0.11; I^2^ = 65%; k = 9). However, the efficacy at follow-up (postpartum period), assessed through a fixed-effect meta-analysis, was not maintained (SMD = −0.16; 95% CI = −0.39, 0.07; I^2^ = 0%; k = 6; Figure 3). A fixed-effect meta-analysis was then carried out to assess the interventions’ efficacy in terms of anxiety symptoms (Figure 4), showing a significant decrease at the intervention endpoint (MD = −1.96; 95% CI = −2.72, −1.21; I^2^ = 0%; k = 3). Subsequently, a random-effect meta-analysis was performed to assess the interventions’ efficacy in terms of sleep quality at the endpoint (Figure 5) and a fixed-effect meta-analysis for sleep quality at follow-up (Figure 6), showing no significant results in either case. 

The interventions’ acceptability evaluated through a random-effect meta-analysis in terms of drop-out rates highlighted that the interventions were well-accepted (OR = 1.68; 95% CI = 0.84, 3.35; I^2^ = 73%; k = 7).

Sensitivity and subgroup analyses were performed through a random-effect meta-analysis, considering depression symptoms as outcome variables and the following as moderators: control group (TAU vs. active), interventions’ theoretical background (CBT vs. third-generation CBT), interventions being based on an intervention full protocol vs. specific techniques or practices, and type of analysis conducted (ITT vs. per-protocol). The results showed that none were significant moderators of the interventions’ efficacy. The specific results of the sub-group analyses, as well as the sensitivity analyses, are reported in tabular form in the Supplementary Materials (Table S5). 

### 3.4. Publication Bias

**Figure 7:**
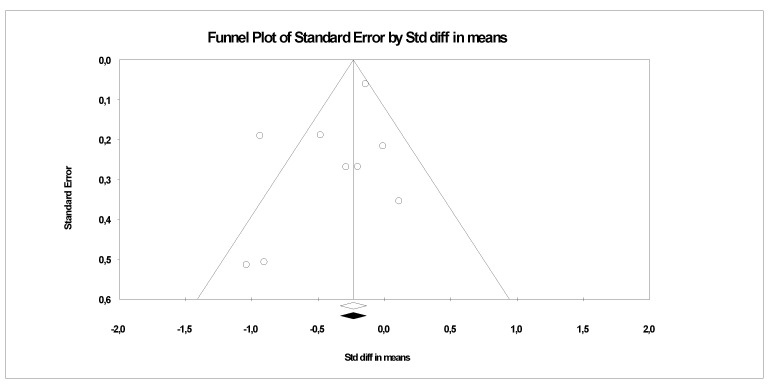
Funnel plots assessing publication bias for depression symptoms.

The publication bias was assessed for depression symptoms, as the outcome was evaluated by all included studies (Figure 7). The Egger regression test was performed, highlighting no publication bias (β0 = 1.23; 1-tail *p* = 0.10). Accordingly, no further analyses were carried out.

### 3.5. Quality Assessment

**Figure 8:**
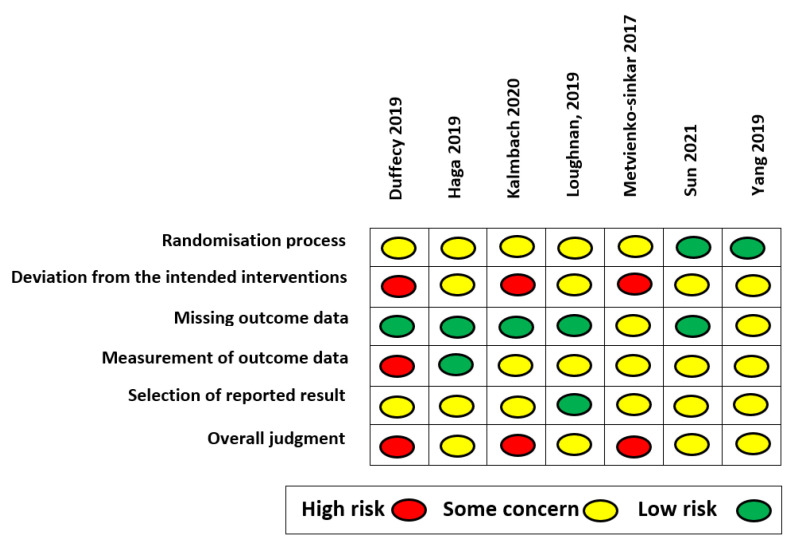
Risk of bias in individual studies.

The quality assessment results for each included RCT are shown in Figure 8. Overall, n = 3 studies showed a high risk of bias (42.86%) and the remaining studies (n = 4) showed some concern for bias (57.14%; see Supplementary Materials, Figure S1). The main source of bias was the deviation from the intended intervention domain (42.86% high risk, 57.14% some concern), followed by the measurement of the outcome data domain (14.29% high risk, 71.43% some concern, 14.29% low risk) and the randomization process domain (71.43% some concern, 28.57% low risk), and lastly the selection of the reported results domain (85.671% some concern, 14.29% low risk). The risk of bias associated with the latter domain was mainly due to the absence of information on the pre-registered analysis to be performed within the studies’ trial registration protocols or the absence of a trial registration protocol. 

## 4. Discussion

The current meta-analysis was aimed at evaluating the overall and differential efficacy of digital CBTs and third-generation CBTs in reducing sub-clinical depressive, anxiety, and stress symptoms while fostering sleep quality and quality of life among pregnant women. 

The results highlighted that most of the included studies relied on third-generation CBTs, in particular on mindfulness practices, to reduce sub-clinical depression and anxiety symptoms. Indeed, among the included studies, the interventions’ efficacy could only be assessed for depression and anxiety symptoms as well as sleep quality. No sufficient data were available on stress symptoms or quality of life, meaning no analysis could be performed. Overall, the results showed no significant improvement in sleep quality, either at the endpoint or at follow-up, yet a significant reduction emerged for depression and anxiety symptoms at the endpoint. The interventions’ efficacy in terms of the anxiety symptoms is in line with the previous evidence on the efficacy of in-person CBTs for perinatal anxiety symptoms [40]. Similarly, the interventions’ efficacy in terms of the depression symptoms is consistent with the findings of a recent meta-analysis focused on digital CBTs administered to perinatal women [41] and a systematic review based on digital CBTs deployed to pregnant women [42]. However, both papers included studies regardless of the women’s psychological symptom level. Moreover, akin to Lau and colleagues’ results [41], the current meta-analysis showed that the efficacy in terms of the depression symptoms was not maintained at follow-up during the postpartum period. Notwithstanding, it should be noted that the focus on depression symptoms compared to the scarce consideration of the highly associated anxiety and stress symptoms, as well as sleep quality and quality of life, represents a limitation of the available literature [50]. Indeed, it is noteworthy that these intervention programs were not specific for women with sub-clinical psychological symptoms during pregnancy. This limitation is of relevance, particularly regarding to the lack of data on quality of life. Indeed, from a prevention standpoint, this gap contrasts with the perspectives delineated by the Constitution of the World Health Organization (WHO), which defines health as “a state of complete physical, mental, and social well-being and not merely the absence of disease or infirmity” (https://apps.who.int/gb/bd/PDF/bd47/EN/constitution-en.pdf?ua=1 (accessed on the 23 March 2022)). Moreover, the absence of evidence-based preventive digital interventions hinders the possibility to further the intents of the stepped-care model [56], thereby limiting the large-scale coordination and scalability of adequate health care services able to support women during the perinatal period.

The moderating role of the interventions’ theoretical background on depression symptoms was also evaluated, showing no significant moderating effect. Accordingly, this result suggests that the efficacy levels of digital traditional vs. third-generation CBTs do not significantly differ in reducing sub-clinical depression symptoms among pregnant women. However, it should be noted that the sensitivity analysis (see Supplementary Materials, Table S5) highlighted that when used alone, neither kind of digital intervention was effective in decreasing depression symptoms, which questions the above-mentioned joint efficacy in terms of the considered symptoms. These results are in line with the findings of a meta-analysis that investigated the efficacy of in-person mindfulness-based interventions (MBIs) on pregnant women, which reported no effect of the MBIs on depression, anxiety, or stress symptoms [44]. These same results, on the other hand, contrast with the above-mentioned meta-analysis reporting the efficacy of CBTs [41], as well as with a recent meta-analysis showing the efficacy of in-person MBIs on perinatal women with sub-clinical symptoms [46]. The latter, in particular, showed a significant intervention effect on pregnant women’s sub-clinical depression symptoms [46]. 

Given the scarce literature collected, it was not possible to evaluate the moderating role of any intervention or sample’s specificities regarding the interventions’ efficacy. In this regard, and mindful of the specificity of the population considered within the current meta-analysis, it might be assumed that by focusing on sub-clinical populations, there are greater individual differences that challenge the development and evaluation of the interventions’ efficacy. Indeed, pregnant women with sub-clinical symptoms differ greatly from each other in their psychosocial functioning and symptomatologic manifestation, thereby being more unique and variable, which represents a challenge when developing intervention and prevention programs, particularly when they are manualized. On the other hand, when pregnant women present clinically relevant symptoms, they tend to be more homogeneous in both their psychosocial functioning and the phenomenological manifestation of suffering, subsuming the common variance that accounts for their shared disorder, leading to the diagnostic categories. Accordingly, interventions and prevention programs should be carefully developed while keeping in mind this variability, which would ultimately support the feasibility and efficacy as regards the interventions’ content soundness.

Taken together, based on the results of the included studies, no sound conclusions on either the joint or differential efficacy of CBT vs. third-generation CBT interventions in supporting pregnant women with sub-clinical psychological symptoms can be drawn. This is most probably due to the limited evidence available as well as the preliminary nature of the current digital psychological interventions, whereby studies were also not sufficiently powered to identify a significant true intervention effect. Referring to the novelty of the field of digital interventions [35,71], a further critical aspect of the reviewed literature is the intervention adherence, for which the user interface and usability are pivotal [72]. Indeed, the quality assessment performed through the risk of bias tool suggested that the studies are of modest to limited quality, particularly regarding the intervention adherence. Although the interventions were deemed acceptable overall, the reduced adherence to the intervention requirements might be a further factor contributing to the impossibility of drawing conclusions regarding the digital interventions’ efficacy. Therefore, future studies designing and developing digital interventions should pay particular attention to the specific features of such interventions, since previous research has stressed the importance of carefully accounting for the user experience quality [73] to support intervention compliance [72]. Moreover, they should ensure that women properly comply with the intervention phases and tasks foreseen as being pivotal in fostering the interventions’ efficacy. A further aspect relevant to usability relates to the specific digital means used to deploy interventions. In the current meta-analysis, all interventions were implemented via web-based platforms, except for two studies [69,70], which instead delivered the intervention through smartphone-based applications. In this regard, albeit during pregnancy women might be more sedentary and better able to comply with the requirements of web-based interventions, which imply a static situation, the added value of smartphone-based interventions is their availability. Indeed, smartphone-based interventions can be available wherever and whenever [74], which might be perceived as being less demanding overall. Furthermore, smartphone-based interventions might have the added value of allowing the collection of ecological data, such as data collected through the ecological momentary assessment methodology [75], which might support the tailoring and adaptability of such interventions in different usage settings [76]. Nonetheless, both smartphone and web-based interventions have their specific benefits and limitations relating to their usability, feasibility, and acceptability, implying a trade-off between these factors. Accordingly, as pointed out by a recent study [74], the choice between a smartphone vs. a web-based deployment approach should be carefully weighted and guided by the specificities of the target population and the intervention aims, requirements, and overall structure [74,77].

The current meta-analysis is though not exempt from limitations. In particular, most of the included studies did not consider a specific range of symptom severity levels but instead included women who had scored from a certain cut-off and onwards (e.g., EPDS > 12). As such, it is not possible to exclude the possibility that a minority of the overall participants considered in the current study representing the right tail of the distribution do present clinically relevant symptoms. 

## 5. Conclusions

To our current knowledge, this is the first meta-analysis that has investigated the overall and differential efficacy levels of digital CBTs and third-generation CBTs in reducing sub-clinical depression, anxiety, and stress symptoms while fostering sleep quality and quality of life among pregnant women. The findings of the present study showed that CBTs and third-generation CBTs, when jointly considered, were effective in decreasing depression symptoms at the intervention endpoint, yet this effect was not maintained at follow-up. However, it is worth noting that the interventions’ effect at the endpoint was not significant when the CBTs and third-generation CBTs were evaluated separately. Regarding the anxiety symptoms during pregnancy, although these symptoms had significantly diminished at the intervention endpoint, these findings were unclear. Moreover, no effect on the stress symptoms emerged, and the interventions’ efficacy in terms of sleep quality and quality of life could not be assessed. Therefore, taken together, no conclusions can be drawn regarding either the joint or differential efficacy of CBT vs. third-generation CBT interventions in reducing sub-clinical depressive, anxiety, and stress symptoms or in supporting the sleep quality and quality of life of pregnant women. Notwithstanding, these digital interventions were overall well-accepted. 

The current meta-analysis was also useful in highlighting the literature limitations that are relevant for future research, thereby highlighting the need for and importance of developing evidence-based digital prevention programs to support pregnant women with sub-clinical psychological symptoms, particularly since the latter tends to be undervalued. More precisely, digital interventions and prevention programs should be greatly tailored to the specificities of the population considered by distinguishing between the antenatal and postnatal periods, as well as by accounting for the individual differences that might confound the findings of significant intervention effects. Furthermore, the interventions’ adherence should be supported by carefully focusing on the user experience quality during the design of these digital solutions. In line with the stepped care approach, greater investment in prevention programs should be provided, thereby favoring the coordination of primary and secondary mental health services [56] and decreasing the costs of the health care system [36,78].

## Figures and Tables

**Table 1:** Sample characteristics.

Author	Country	N		Age M (SD)		Marital Status N; %		Educational Level N; %		Weeks Pregnant M (SD)	
		EG	CG	EG	CG	EG	CG	EG	CG	EG	CG
[64]	USA	18	7	30.5 (4.05)		Married or cohabitating 20; 83%		Graduate or post-graduate 25; 100%		n/r	n/r
[65]	Norway	678	664	31 (4.6)	31.1 (4.5)	n/r	n/r	≤high school 100, 14.7% 1–3 years of university 189, 27.9% ≥4–5 years college or university 389, 57.4%	≤high school 107, 16.1% 1–3 years of university 183, 27.6% ≥4–5 years college or university 374, 56.3%	n/r	n/r
[66]	USA	46	45	28.91 (4.25)	29.16 (4.11)	n/r	n/r	n/r	n/r	n/r	n/r
[67]	Australia	36	41	31.69 (4.44)	31.54 (3.63)	In a relationship 10; 28% Separated or divorced 1; 3% Married 23; 64% Single 2; 6%	In a relationship 4; 10% Separated or divorced 0; 0% Married 36; 88% Single 1; 2%	<high-school 3; 8% Trace certificate/Diploma 8; 22% Undergraduate degree 17; 47% Post-graduate degree 8; 22%	<high-school 1; 2% Trace certificate/Diploma 4; 10% Undergraduate degree 29; 71% Post-graduate degree 7; 17%	20.54 (6.01)	22.63 (5.76)
[68] #	Ireland	32	14	33.81(2.53)		In a relationship 4; 11.1% Married 32; 88.9%		n/r	n/r	16.15 (2.88)	
[69]	China	84	84	30.27 (3.8)	29.55 (4.21)	Married 167; 100%		n/r	n/r	13.81 * (na)	14.41 * (na)
[70]	China	62	61	31.31 (4.97)	30.98 (3.91)	n/r	n/r	≤high-school 5; 8.1% Some college 19; 30.6% Undergraduate degree 31; 50% ≥Post-graduate degree 7; 11.3%	≤high-school 11; 18% Some college 15; 24.6% Undergraduate degree 27; 44.3% ≥Post-graduate degree 8; 13.1%	25.52 (1.84)	26.33 (3.45)

Note. EG = experimental group; CG = control group; n/r = not reported; na = not available; # data refer only to participants who had completed the intervention; * in the original study the data were reported as days, while here they were calculated and reported as weeks by dividing them by 7.

**Table 2:** Intervention characteristics.

Author	Intervention	Control Condition	Theoretical Background	Full-Protocol vs. Techniques	Length (Weeks)	Intervention Description
[64]	*Share* Web-based	Active; same as *Share* without the group activities	CBT	Techniques	8	Unguided group intervention that comprised 16 main didactic activities. It provided information on both pregnancy and the postpartum period. The intervention also included 3 postpartum bust sessions (not considered in the current study). The CBT techniques employed were the following: (1) thought restructuring; (2) mood tracking; (3) activity scheduling; (4) monitoring; (5) relaxation; (6) goal setting. Participants could also interact with other women (by posting, leaving comments or likes on their posts, and by sending direct messages) encouraging discussion among them after each lesson. Participants had a personal profile in which they could post information about themselves to increase group bonding.
[65]	Mamma Mia * Web-based	TAU	Third generation CBT	Techniques	16 #	An unguided program that included 11 sessions each, which needed to be concluded to proceed to the following lesson. Provided psychoeducational information on pregnancy in a step-by-step fashion as well as cognitive and behavioral assignments. The intervention was focused on the following: (1) providing information on the specific perinatal period; (2) working on expectancies and attitudes; (3) supporting attachment, emotion regulation, and help-seeking; (4) working on relationship satisfaction and communication skills.
[66]	*Sleepio* Web-based CBTI program	Active; sleep education	CBT	Full protocol	6	Digital CBT intervention was specific for insomnia symptoms and included 6 sessions. The sessions were guided by a personal agent or “virtual therapist.” New sessions could be completed only after finishing the preceding one. The interventions included: (1) behavioral components (sleep restriction, stimulus control); (2) cognitive components (e.g., cognitive restructuring, paradoxical intention); (3) progressive muscle relaxation; (4) sleep hygiene.
[67]	*MUMmentum* Web-based	TAU	CBT	Full protocol	4	Unguided CBT intervention specific for pregnant women showing generalized anxiety and depression symptoms. It included three lessons during which content was presented through illustrated stories displayed using slides to learn how to self-manage anxiety and depression symptoms. Overall the intervention included: (1) psychoeducation; (2) relaxation techniques; (3) thought challenging; (4) structured problem solving; (5) active planning and monitoring; (6) grade exposure; (7) assertive communication; (8) relapse prevention; (9) sleep hygiene; (10) pleasant activities; (11) self-care plans; (10) understanding intrusive thoughts and images.
[68]	*Gratitude and Mindfulness* Web-based	TAU	Third generation CBT	Techniques	3	The unguided intervention focused on two main components: (1) a gratitude diary that was aimed at favoring reflection on the pregnancy experience; (2) mindfulness audio tracks, particularly the body scan practice, during which the focus was placed on breathing and on paying attention to each part of the body.
[69]	*Spirits Healing* Smartphone-based	Active; attention control group	Third generation CBT	Full protocol	8	Revised unguided MBCT focused on perinatal depression and negative emotions and on supporting the adaptation to the body changes given by the pregnancy. It included formal mindfulness training deployed through videos, reading material, and audio tracks for guided mindfulness practices. It comprised 8 sessions focused on: (1) providing information on mindfulness; (2) increasing focus on the present; (3) supporting mindfulness of negative emotions; (4) accepting difficulties; (5) understanding that thoughts are only thoughts; (6) supporting the enjoyment of daily happiness; (7) favoring mindfulness during pregnancy and childbirth; (8) continuing mindfulness practice.
[70]	*Mindfulness intervention* Smartphone-based	TAU	Third generation CBT	Techniques	8	Unguided mindfulness intervention was created ad hoc by a multidisciplinary team and supported by mindfulness-trained nurses who provided technical help while monitoring for changes in the symptom levels. Four mindfulness sessions were video-recorded by the mindfulness-trained nurses and shared on a smartphone chat platform (Wechat) and supplemented by text, pictures, and audio recordings that women could review. These sessions were focused on different mindfulness practices: (1) body screening; (2) relaxation; (3) meditation. During each session, the participants reviewed what had been done in the previous session and were then introduced to new mindfulness constructs. Between-session homework was foreseen by the intervention.

Note. * = Since the intervention was structured in three phases beginning during pregnancy and terminating during postpartum, the present study only considered the first phase administered during pregnancy. ^#^ = approximation: the intervention started during either the 21st or 25th gestational week and terminated during the 37th gestational week for each participant.

## Supplementary Materials

The following supporting information can be downloaded at: https://www.mdpi.com/article/10.3390/ijerph19159549/s1. Figure S1: Overall risk of bias results shown as percentages. Table S1: PRISMA 2020 checklist. Table S2: Excluded studies with reasons. Table S3: Study sample characteristics. Table S4: Outcome characteristics. Table S5: Sub-group analysis using a fixed-effect meta-analysis.
