# Supplementary material for: The Efficacy of Digital Cognitive–Behavioral Interventions in Supporting the Psychological Adjustment and Sleep Quality of Pregnant Women with Sub-Clinical Symptoms: A Systematic Review and Meta-Analysis
Source: Int J Environ Res Public Health. 2022 Aug 3;19(15):9549. doi: 10.3390/ijerph19159549 (PMC9368246; doi:10.3390/ijerph19159549)
Supplement: Supplementary file 1 [file ijerph-19-09549-s001.zip › ijerph-1753918-supplementary.pdf]

Table S1. PRISMA 2020 Checklist

PRISMA 2020 Checklist

Section andTopic	Item#	Checklist item	Location where item is reported
**TITLE**			
Title	1	Identify the report as a systematic review.	1
**ABSTRACT**			
Abstract	2	See the PRISMA 2020 for Abstracts checklist.	1
**INTRODUCTION**			
Rationale	3	Describe the rationale for the review in the context of existing knowledge.	1-3
Objectives	4	Provide an explicit statement of the objective(s) or question(s) the review addresses.	2-3
**METHODS**			
Eligibility criteria	5	Specify the inclusion and exclusion criteria for the review and how studies were grouped for the syntheses.	3
Informatio nsources	6	Specify all databases, registers, websites, organisations, reference lists and other sources searched or consulted to identify studies. Specify thedate when each source was last searched or consulted.	3-4
Search strategy	7	Present the full search strategies for all databases, registers and websites, including any filters and limits used.	3-4
Selection process	8	Specify the methods used to decide whether a study met the inclusion criteria of the review, including how many reviewers screened each recordand each report retrieved, whether they worked independently, and if applicable, details of automation tools used in the process.	4
Data collection process	9	Specify the methods used to collect data from reports, including how many reviewers collected data from each report, whether they worked independently, any processes for obtaining or confirming data from study investigators, and if applicable, details of automation tools used in theprocess.	4
Data items	10a	List and define all outcomes for which data were sought. Specify whether all results that were compatible with each outcome domain in each study were sought (e.g. for all measures, time points, analyses), and if not, the methods used to decide which results to collect.	3-4
	10b	List and define all other variables for which data were sought (e.g. participant and intervention characteristics, funding sources). Describe any assumptions made about any missing or unclear information.	3-4
Study risk of bias assessment	11	Specify the methods used to assess risk of bias in the included studies, including details of the tool(s) used, how many reviewers assessed each study and whether they worked independently, and if applicable, details of automation tools used in the process.	4
Effect measures	12	Specify for each outcome the effect measure(s) (e.g. risk ratio, mean difference) used in the synthesis or presentation of results.	4
Synthesi s methods	13a	Describe the processes used to decide which studies were eligible for each synthesis (e.g. tabulating the study intervention characteristics andcomparing against the planned groups for each synthesis (item #5)).	4-5
	13b	Describe any methods required to prepare the data for presentation or synthesis, such as handling of missing summary statistics, or dataconversions.	4-5

	13c	Describe any methods used to tabulate or visually display results of individual studies and syntheses.	4-5
	13d	Describe any methods used to synthesize results and provide a rationale for the choice(s). If meta-analysis was performed, describe the model(s), method(s) to identify the presence and extent of statistical heterogeneity, and software package(s) used.	4-5
	13e	Describe any methods used to explore possible causes of heterogeneity among study results (e.g. subgroup analysis, meta-regression).	4-5
	13f	Describe any sensitivity analyses conducted to assess robustness of the synthesized results.	4-5
Reporting bias assessment	14	Describe any methods used to assess risk of bias due to missing results in a synthesis (arising from reporting biases).	Na
Certainty assessme nt	15	Describe any methods used to assess certainty (or confidence) in the body of evidence for an outcome.	4-5
**RESULTS**			
Study selection	16a	Describe the results of the search and selection process, from the number of records identified in the search to the number of studies included in the review, ideally using a flow diagram.	5
	16b	Cite studies that might appear to meet the inclusion criteria, but which were excluded, and explain why they were excluded.	Supplementary Materials Table S2
Study characteristic s	17	Cite each included study and present its characteristics.	5-10 and Supplementary Materials Table S3 and Table S4
Risk of bias instudies	18	Present assessments of risk of bias for each included study.	13
Results of individual studies	19	For all outcomes, present, for each study: (a) summary statistics for each group (where appropriate) and (b) an effect estimate and its precision(e.g. confidence/credible interval), ideally using structured tables or plots.	11-12
Results of synthese s	20a	For each synthesis, briefly summarise the characteristics and risk of bias among contributing studies.	11-13
	20b	Present results of all statistical syntheses conducted. If meta-analysis was done, present for each the summary estimate and its precision (e.g.confidence/credible interval) and measures of statistical heterogeneity. If comparing groups, describe the direction of the effect.	11-12
	20c	Present results of all investigations of possible causes of heterogeneity among study results.	11 and Supplementary Materials Table S5
	20d	Present results of all sensitivity analyses conducted to assess the robustness of the synthesized results.	Supplementary Materials Table S5
Reporting biases	21	Present assessments of risk of bias due to missing results (arising from reporting biases) for each synthesis assessed.	Supplementary Materials Figure S1
Certainty of evidence	22	Present assessments of certainty (or confidence) in the body of evidence for each outcome assessed.	12
**DISCUSSION**			

Discussion	23a	Provide a general interpretation of the results in the context of other evidence.	13-15
	23b	Discuss any limitations of the evidence included in the review.	13-15
	23c	Discuss any limitations of the review processes used.	15
	23d	Discuss implications of the results for practice, policy, and future research.	13-15
**OTHER INFORMATION**			
Registration andprotocol	24a	Provide registration information for the review, including register name and registration number, or state that the review was not registered.	3
	24b	Indicate where the review protocol can be accessed, or state that a protocol was not prepared.	3
	24c	Describe and explain any amendments to information provided at registration or in the protocol.	Na
Support	25	Describe sources of financial or non-financial support for the review, and the role of the funders or sponsors in the review.	16
Competin g interests	26	Declare any competing interests of review authors.	16
Availability of data, code andother materials	27	Report which of the following are publicly available and where they can be found: template data collection forms; data extracted from includedstudies; data used for all analyses; analytic code; any other materials used in the review.	na

Table S2. Excluded studies with reason

Author	Exclusion reason
[1]	Not a digital intervention; include participants younger than 18 years old
[2]	Not a digital intervention; Not an RCT
[3]	Not an RCT
[4]	Not an RCT
[5]	Included women with a history of Major depression; The study does not report the data necessary to conduct analysis
[6]	Included women with a history of Major depression; Not a digital intervention
[7]	Study protocol; Not a digital intervention
[8]	Not a digital intervention; includes participants with psychiatry history
[9]	Not a digital intervention; includes participants with psychiatry history
[10]	Not a digital intervention
[11]	Not an RCT; Not a digital intervention
[12]	Not a digital intervention; Not an RCT; Includes participants with psychiatry history
[13]	Not a digital intervention; Includes participants with psychiatry history
[14]	Not a digital intervention
[15]	Included both pregnant women and women in the post-partum period
[16]	Includes participants with psychiatry history; Not an RCT
[17]	Incudes women with insomnia disorder
[18]	Included both pregnant women and women in the post-partum period as well as women with a psychiatry history
[19]	Include women with major depression
[20]	Included women in the post-partum period
[21]	The necessary data to conduct analysis are not reported
[22]	Does not include any of our variables of interest
[23]	Not a digital intervention
[24]	Not an RCT; Not a CBT or 3rd Generation CBT intervention, nor grounded on them
[25]	Not a CBT or 3rd Generation CBT intervention, nor grounded on them
[26]	Include women with current depressive and/or anxiety disorder and that were undergoing psychological and/or psychotropic treatment
[27]	Not a digital intervention
[28]	Not an RCT
[29]	Included currently pregnant, pregnant within the last year and intending to become pregnant women.
[30]	Not a CBT or 3rd Generation CBT intervention, nor grounded on them
[31]	Not an RCT
[32]	Included women with current psychiatric history
[33]	Not an RCT
[34]	Not a digital intervention
[35]	Not a digital intervention; Include women with major depression
[36]	Not a digital intervention
[37]	Not a digital intervention
[38]	Not a digital intervention
[39]	Included women in the post-partum period; included women meeting criteria for major depression or post-partum depression; The intervention is not CBT or 3rd Generation CBT, nor grounded on them
[40]	Not a digital intervention

[41]	Not a CBT or 3rd Generation CBT intervention, nor grounded on them
[42]	Compared two CBT interventions
[43]	Not an RCT; Not CBT or 3rd Generation CBT
[44]	Does not include pregnant women; Not CBT or 3rd Generation CBT, nor grounded on them
[45]	Not a digital intervention
[46]	Not a CBT or 3rd Generation CBT intervention, nor grounded on them
[47]	Not a digital intervention
[48]	Not a digital intervention; Includes women meeting criteria for major depression disorder

Table S3. Studies' sample characteristics

Author	N		Ethnicity N; %		Working status N; %		Previous Pregnancies N; %	
	EG	EG	EG	CG	EG	CG	EG	CG
[49]	18	7	n/r		▪ Employed 16; 67%<br>▪ Unemployed 8; 33%		n/r	
[50]	678	664	n/r		n/r		▪ Nulliparous 393; 58%<br>▪ Multiparous 285; 42%	▪ Nulliparous 382; 57,7%<br>▪ ≤ Multiparous 282; 42,5%
[51]	46	45	▪ White 23; 51,1%<br>▪ Black 14; 31,1%<br>▪ Asian 3; 6,7%<br>▪ Middle Eastern 2; 4,4%<br>▪ Hispanic/Latino 1; 2,2%<br>▪ Multiracial 2; 4,4%	▪ White 24; 52,2%<br>▪ Black 15; 32,6%<br>▪ Asian 3; 5%<br>▪ Middle Eastern 2; 4,3%<br>▪ Hispanic or Latino 1; 2,2%<br>▪ Multiracial 1; 2,2%	n/r		n/r	
[52]	36	41	n/r		▪ Full-time 19; 52,78%<br>▪ Part-time 11; 30,56%<br>▪ Student 4; 11,11%<br>▪ Homemaker2; 5,56%<br>▪ Unemployed 1; 2,78%	▪ Full-time 21; 51,22%<br>▪ Part-time 13; 31,71%<br>▪ Student 2; 4,88%<br>▪ Homemaker5; 12,2%<br>▪ Unemployed 0; 0%	▪ Nulliparous 10; 28%<br>▪ Multiparous 26; 72%	▪ Nulliparous 15; 37%<br>▪ Multiparous 26; 63%
[53]	32	14	n/r		n/r		n/r	
[54]	84	84	▪ Chinese Han 84; 100%<br>▪ Chinese Hui 0;0%	▪ Chinese Han 83; 99%<br>▪ Chinese Hui 1; 1%	▪ Employed 67; 81%<br>▪ Unemployed 16; 19%	▪ Employed 58; 72%<br>▪ Unemployed 22; 28%	n/r	
[55]	62	61	n/r		▪ Employed 47; 75.8%<br>▪ Unemployed 15; 24.2%	▪ Employed 40; 65.6<br>▪ Unemployed 21; 34.4	▪ Nulliparous 39; 62.9%<br>▪ Multiparous 23; 37.1%	▪ Nulliparous 38; 62.3%<br>▪ Multiparous 23; 37.7%

Table S4. Outcomes' characteristics

Author	Outcomes of interest	Measurement tool	Single study's inclusion cut-off	Follow-up	
[49]	▪ Depression	▪ PHQ-9*<br>▪ HDRS*	PHQ-8: range 5 - 14	▪ PHQ-9<br>▪ HDRS	6 weeks post-partum
[50]	▪ Depression	▪ EPDS*	EPDS ≥ 10	na	
[51]	▪ Depression<br>▪ Sleep quality	▪ EPDS*<br>▪ ISI* & PSQI*	ISI ≥ 10	▪ EPDS<br>▪ ISI & PSQI	6 weeks post-partum
[52]	▪ Depression<br>▪ Anxiety<br>▪ Quality of Life	▪ EPDS* & PHQ-9*<br>▪ GAD-7<br>▪ WHQOL-Bref	PHQ-9 > 9<br>or<br>GAD > 9	▪ EPDS & PHQ-9<br>▪ GAD-7<br>▪ WHQOL-Bref	4 weeks post-partum
[53]	▪ Depression<br>▪ Stress	▪ EPDS*<br>▪ PDQ	na	na	
[54]	▪ Depression<br>▪ Anxiety<br>▪ Stress<br>▪ Sleep Quality	▪ EPDS* & PHQ-9#<br>▪ GAD-7<br>▪ PSS<br>▪ PSQI*	PHQ-9 > 4<br>or<br>EPDS > 9	▪ EPDS<br>▪ GAD-7<br>▪ PSS<br>▪ PSQI*	6 weeks post-partum
[55]	▪ Depression<br>▪ Anxiety	▪ PHQ-9<br>▪ GAD-7	PHQ-9 range 4 - 14<br>and<br>GAD-7 range 4 – 14	na	

Note. * = considered in the analysis; # = considered by the study's Authors only to assess participants' eligibility; GAD-7 = Generalized Anxiety Disorder-7; EPDS = Edinburgh Postnatal Depression Scale; ISI = Insomnia Severity Index; HDRS = Hamilton Depression Rating Scale;PHQ-8 = Patient Health Questionnaire – 8; PHQ-9 = Patient Health Questionnaire – 9; PSS = Perceived Stress Scale; PSQI = Pittsburgh Sleep Quality Index; PDQ = Prenatal Distress Questionnaire; WHOQOL-Bref = WHO Quality of Life-BREF.

Table S5. Sub-group and sensitivity analysis results using fixed-effect meta-analysis – Depression symptoms

Depression symptoms					
	k	SMD	95% CI	I^2	*p*
Control condition					
TAU	5	-0.32	-0.67, 0.04	76%	0.70
Active control	4	-0.46	-0.83, -0.02	45%	
Theoretical Background					
CBT	5	-0.27	-0.57, 0.02	16%	0.65
Third-generation CBT	4	-0.36	-0.81, 0.02	65%	
Full-Protocol vs. Techniques					
Full-Protocol	4	-0.27	-0.49, -0.04	0%	0.40
Techniques	5	-0.51	-1.00, -0.01	65%	
Type of analysis					
ITT	6	-0.34	-0.62, -0.07	73%	0.69
Per-protocol	3	-0.50	-1.25, 0.24	52%	

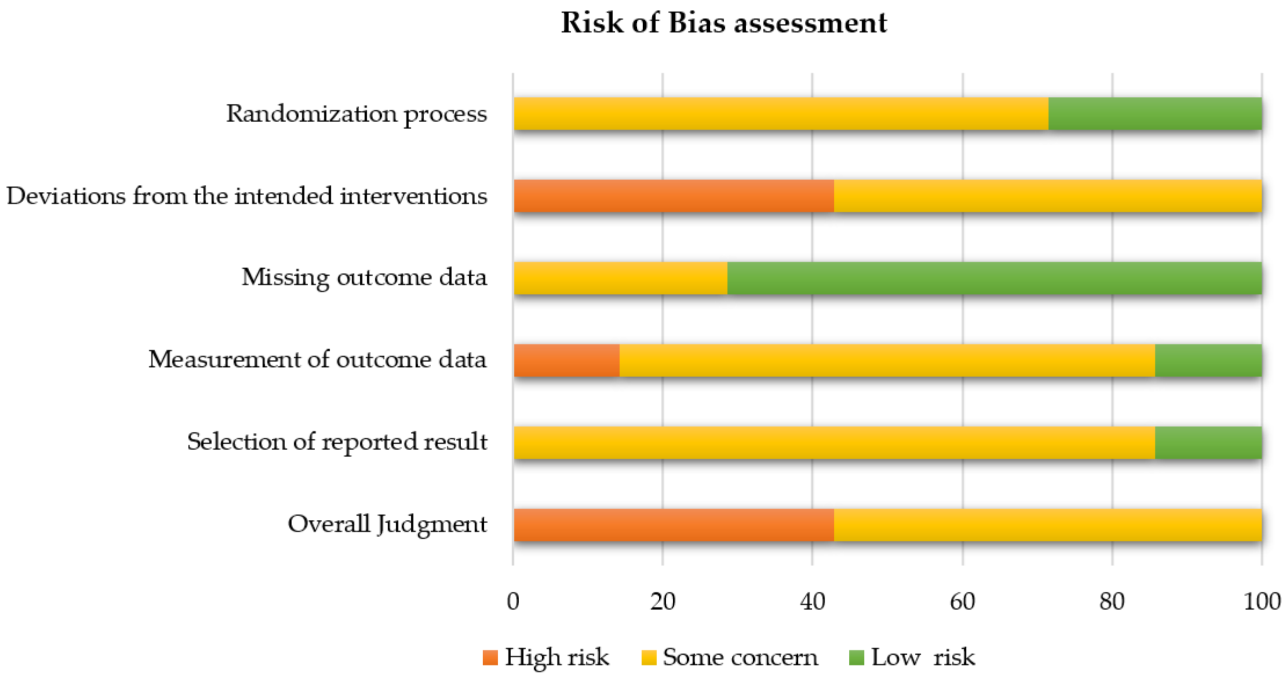

Figure S1. Overall Risk of Bias results shown as percentages.

Bibliography

1. Alhusen, J.L.; Hayat, M.J.; Borg, L. A Pilot Study of a Group-Based Perinatal Depression Intervention on Reducing Depressive Symptoms and Improving Maternal-Fetal Attachment and Maternal Sensitivity. *Arch Womens Ment Health* **2021**, *24*, 145–154, doi:10.1007/s00737-020-01032-0.
2. Atif, N.; Nazir, H.; Zafar, S.; Chaudhri, R.; Atiq, M.; Mullany, L.C.; Rowther, A.A.; Malik, A.; Surkan, P.J.; Rahman, A. Development of a Psychological Intervention to Address Anxiety During Pregnancy in a Low-Income Country. *Front Psychiatry* **2020**, *10*, 927, doi:10.3389/fpsyt.2019.00927.
3. Barber, C.; Clark, M.; Williams, S.; Isler, R. Relaxation and Mindfulness to Manage Stress in Pregnancy: Initial Studies of a Computerised Self-Help Programme. **2013**.
4. Barber, C.C.; Masters-Awatere, B. Positively Pregnant: Development and Piloting of a Mobile App for Social and Emotional Well-Being in Pregnancy. *Applied Psychology: Health and Well-Being n/a*, doi:10.1111/aphw.12333.
5. Barrera, A.Z.; Wickham, R.E.; Muñoz, R.F. Online Prevention of Postpartum Depression for Spanish- and English-Speaking Pregnant Women: A Pilot Randomized Controlled Trial. *Internet Interventions* **2015**, *2*, 257–265, doi:10.1016/j.invent.2015.06.002.
6. Beck, A.; Dimidjian, S.; Sherwood, N.; Goodman, S.; Welch, S.; Ludman, E.; Boggs, J.; Metcalf, C.; Simon, G. C4-5: Behavioral Activation Therapy for Perinatal Depression: Preliminary Results from a Multi-Site Randomized Trial. *Clinical Medicine & Research* **2014**, *12*, 103–103, doi:10.3121/cmr.2014.1250.c4-5.
7. Bei, B.; Pinnington, D.M.; Shen, L.; Blumfield, M.; Drummond, S.P.A.; Newman, L.K.; Manber, R. A Scalable Cognitive Behavioural Program to Promote Healthy Sleep during Pregnancy and Postpartum Periods: Protocol of a Randomised Controlled Trial (the SEED Project). *BMC Pregnancy Childbirth* **2019**, *19*, 254, doi:10.1186/s12884-019-2390-8.
8. Bittner, A.; Peukert, J.; Zimmermann, C.; Junge-Hoffmeister, J.; Parker, L.S.; Stöbel-Richter, Y.; Weidner, K. Early Intervention in Pregnant Women With Elevated Anxiety and Depressive Symptoms: Efficacy of a Cognitive-Behavioral Group Program. *Journal of Perinatal & Neonatal Nursing* **2014**, *28*, 185–195, doi:10.1097/JPN.0000000000000027.
9. Burger, H.; Verbeek, T.; Aris-Meijer, J.L.; Beijers, C.; Mol, B.W.; Hollon, S.D.; Ormel, J.; van Pampus, M.G.; Bockting, C.L.H. Effects of Psychological Treatment of Mental Health Problems in Pregnant Women to Protect Their Offspring: Randomised Controlled Trial. *Br J Psychiatry* **2020**, *216*, 182–188, doi:10.1192/bjp.2019.260.
10. Byatt, N.; Moore Simas, T.A.; Biebel, K.; Sankaran, P.; Pbert, L.; Weinreb, L.; Ziedonis, D.; Allison, J. PRogram In Support of Moms (PRISM): A Pilot Group Randomized Controlled Trial of Two Approaches to Improving Depression among Perinatal Women. *Journal of Psychosomatic Obstetrics & Gynecology* **2018**, *39*, 297–306, doi:10.1080/0167482X.2017.1383380.
11. Dimidjian, S.; Goodman, S.H. Preferences and Attitudes toward Approaches to Depression Relapse/Recurrence Prevention among Pregnant Women. *Behaviour Research and Therapy* **2014**, *54*, 7–11, doi:10.1016/j.brat.2013.11.008.
12. Dimidjian, S.; Goodman, S.H.; Felder, J.N.; Gallop, R.; Brown, A.P.; Beck, A. An Open Trial of Mindfulness-Based Cognitive Therapy for the Prevention of Perinatal Depressive Relapse/Recurrence. *Arch Womens Ment Health* **2015**, *18*, 85–94, doi:10.1007/s00737-014-0468-x.
13. Dimidjian, S.; Goodman, S.H.; Felder, J.N.; Gallop, R.; Brown, A.P.; Beck, A. Staying Well during Pregnancy and the Postpartum: A Pilot Randomized Trial of Mindfulness-Based Cognitive Therapy for the Prevention of Depressive Relapse/Recurrence. *Journal of Consulting and Clinical Psychology* **2016**, *84*, 134–145, doi:10.1037/ccp0000068.
14. Dimidjian, S.; Goodman, S.H.; Sherwood, N.E.; Simon, G.E.; Ludman, E.; Gallop, R.; Welch, S.S.; Boggs, J.M.; Metcalf, C.A.; Hubley, S.; et al. A Pragmatic Randomized Clinical Trial of Behavioral Activation for Depressed Pregnant Women. *Journal of Consulting and Clinical Psychology* **2017**, *85*, 26–36, doi:10.1037/ccp0000151.

15. Felder, J.N. TARGETING BARRIERS TO CARE FOR PREGNANT WOMEN AT RISK FOR DEPRESSION: EXAMINING THE ROLE OF STIGMA AND THE FEASIBILITY OF A WEB-BASED DEPRESSION PREVENTION PROGRAM. 102.
16. Felder, J.N.; Segal, Z.; Beck, A.; Sherwood, N.E.; Goodman, S.H.; Boggs, J.; Lemon, E.; Dimidjian, S. An Open Trial of Web-Based Mindfulness-Based Cognitive Therapy for Perinatal Women at Risk for Depressive Relapse. *Cognitive and Behavioral Practice* **2017**, *24*, 26–37, doi:10.1016/j.cbpra.2016.02.002.
17. Felder, J.N.; Epel, E.S.; Neuhaus, J.; Krystal, A.D.; Prather, A.A. Efficacy of Digital Cognitive Behavioral Therapy for the Treatment of Insomnia Symptoms Among Pregnant Women: A Randomized Clinical Trial. *JAMA Psychiatry* **2020**, *77*, 484, doi:10.1001/jamapsychiatry.2019.4491.
18. Fonseca, A.; Monteiro, F.; Alves, S.; Gorayeb, R.; Canavarro, M.C. Be a Mom, a Web-Based Intervention to Prevent Postpartum Depression: The Enhancement of Self-Regulatory Skills and Its Association With Postpartum Depressive Symptoms. *Front. Psychol.* **2019**, *10*, 265, doi:10.3389/fpsyg.2019.00265.
19. Forsell, E.; Bendix, M.; Holländare, F.; Szymanska von Schultz, B.; Nasiell, J.; Blomdahl-Wetterholm, M.; Eriksson, C.; Kvarned, S.; Lindau van der Linden, J.; Söderberg, E.; et al. Internet Delivered Cognitive Behavior Therapy for Antenatal Depression: A Randomised Controlled Trial. *Journal of Affective Disorders* **2017**, *221*, 56–64, doi:10.1016/j.jad.2017.06.013.
20. Gold, K.J.; Boggs, M.E.; Kavanaugh, K.L. MOMSonLINE: Lessons Learned From a Feasibility RCT of Online Support for Mothers Bereaved by Perinatal Loss. *Omega (Westport)* **2021**, *83*, 656–672, doi:10.1177/0030222819861558.
21. Guo, L.; Zhang, J.; Mu, L.; Ye, Z. Preventing Postpartum Depression With Mindful Self-Compassion Intervention: A Randomized Control Study. *J Nerv Ment Dis* **2020**, *208*, 101–107, doi:10.1097/NMD.0000000000001096.
22. Haga, S.M.; Kinser, P.; Wentzel-Larsen, T.; Lisøy, C.; Garthus-Niegel, S.; Slinning, K.; Drozd, F. Mamma Mia – A Randomized Controlled Trial of an Internet Intervention to Enhance Subjective Well-Being in Perinatal Women. *The Journal of Positive Psychology* **2021**, *16*, 446–454, doi:10.1080/17439760.2020.1738535.
23. Hamilton, J.; Saxon, D.; Best, E.; Glover, V.; Walters, S.J.; Kerr, I.B. A Randomized, Controlled Pilot Study of Cognitive Analytic Therapy for Stressed Pregnant Women with Underlying Anxiety and Depression in a Routine Health Service Setting. *Clin Psychol Psychother* **2021**, *28*, 394–408, doi:10.1002/cpp.2520.
24. Hantsoo, L.; Podcasy, J.; Sammel, M.; Epperson, C.N.; Kim, D.R. Pregnancy and the Acceptability of Computer-Based Versus Traditional Mental Health Treatments. *Journal of Women's Health* **2017**, *26*, 1106–1113, doi:10.1089/jwh.2016.6255.
25. Hantsoo, L.; Criniti, S.; Khan, A.; Moseley, M.; Kincler, N.; Faherty, L.J.; Epperson, C.N.; Bennett, I.M. A Mobile Application for Monitoring and Management of Depressed Mood in a Vulnerable Pregnant Population. *PS* **2018**, *69*, 104–107, doi:10.1176/appi.ps.201600582.
26. Heller, H.M.; Hoogendoorn, A.W.; Honig, A.; Broekman, B.F.P.; van Straten, A. The Effectiveness of a Guided Internet-Based Tool for the Treatment of Depression and Anxiety in Pregnancy (MamaKits Online): Randomized Controlled Trial. *J Med Internet Res* **2020**, *22*, e15172, doi:10.2196/15172.
27. Jesse, D.E.; Gaynes, B.N.; Feldhousen, E.B.; Newton, E.R.; Bunch, S.; Hollon, S.D. Performance of a Culturally Tailored Cognitive-Behavioral Intervention Integrated in a Public Health Setting to Reduce Risk of Antepartum Depression: A Randomized Controlled Trial. *Journal of Midwifery & Women's Health* **2015**, *60*, 578–592, doi:10.1111/jmwh.12308.
28. Kalmbach, D.A.; Cheng, P.; Roth, T.; Swanson, L.M.; Cuamatzi-Castelan, A.; Roth, A.; Drake, C.L. Examining Patient Feedback and the Role of Cognitive Arousal in Treatment Non-Response to Digital Cognitive-Behavioral Therapy for Insomnia during Pregnancy. *Behavioral Sleep Medicine* **2022**, *20*, 143–163, doi:10.1080/15402002.2021.1895793.
29. Kelman, A.R.; Evare, B.S.; Barrera, A.Z.; Muñoz, R.F.; Gilbert, P. A Proof-of-Concept Pilot Randomized Comparative Trial of Brief Internet-Based Compassionate Mind Training and Cognitive-Behavioral Therapy for Perinatal and Intending to Become Pregnant Women. *Clin Psychol Psychother* **2018**, *25*, 608–619, doi:10.1002/cpp.2185.

30. Kingston, D.; Austin, M.-P.; Veldhuyzen van Zanten, S.; Harvalik, P.; Giallo, R.; McDonald, S.D.; MacQueen, G.; Vermeyden, L.; Lasiuk, G.; Sword, W.; et al. Pregnant Women's Views on the Feasibility and Acceptability of Web-Based Mental Health E-Screening Versus Paper-Based Screening: A Randomized Controlled Trial. *J Med Internet Res* **2017**, *19*, e88, doi:10.2196/jmir.6866.
31. Kinser, P.A.; Thacker, L.R.; Rider, A.; Moyer, S.; Amstadter, A.B.; Mazzeo, S.E.; Bodnar-Deren, S.; Starkweather, A. Feasibility, Acceptability, and Preliminary Effects of "Mindful Moms": A Mindful Physical Activity Intervention for Pregnant Women with Depression. *Nursing Research* **2021**, *70*, 95–105, doi:10.1097/NNR.0000000000000485.
32. Krusche, A.; Dymond, M.; Murphy, S.E.; Crane, C. Mindfulness for Pregnancy: A Randomised Controlled Study of Online Mindfulness during Pregnancy. *Midwifery* **2018**, *65*, 51–57, doi:10.1016/j.midw.2018.07.005.
33. Kubo, A.; Aghaee, S.; Kurtovich, E.M.; Nkemere, L.; Quesenberry, C.P.; McGinnis, M.K.; Avalos, L.A. MHealth Mindfulness Intervention for Women with Moderate-to-Moderately-Severe Antenatal Depressive Symptoms: A Pilot Study Within an Integrated Health Care System. *Mindfulness* **2021**, *12*, 1387–1397, doi:10.1007/s12671-021-01606-8.
34. Le, H.-N.; Perry, D.F.; Stuart, E.A. Randomized Controlled Trial of a Preventive Intervention for Perinatal Depression in High-Risk Latinas. *Journal of Consulting and Clinical Psychology* **2011**, *79*, 135–141, doi:10.1037/a0022492.
35. Lenze, S.N.; Potts, M.A. Brief Interpersonal Psychotherapy for Depression during Pregnancy in a Low-Income Population: A Randomized Controlled Trial. *Journal of Affective Disorders* **2017**, *210*, 151–157, doi:10.1016/j.jad.2016.12.029.
36. MacKinnon, A.L.; Madsen, J.W.; Giesbrecht, G.F.; Campbell, T.; Carlson, L.E.; Dimidjian, S.; Letourneau, N.; Tough, S.; Tomfohr-Madsen, L. Effects of Mindfulness-Based Cognitive Therapy in Pregnancy on Psychological Distress and Gestational Age: Outcomes of a Randomized Controlled Trial. *Mindfulness* **2021**, *12*, 1173–1184, doi:10.1007/s12671-020-01585-2.
37. Manber, R.; Bei, B.; Simpson, N.; Asarnow, L.; Rangel, E.; Sit, A.; Lyell, D. Cognitive Behavioral Therapy for Prenatal Insomnia: A Randomized Controlled Trial. *Obstetrics & Gynecology* **2019**, *133*, 911–919, doi:10.1097/AOG.0000000000003216.
38. Milgrom, J.; Schembri, C.; Ericksen, J.; Ross, J.; Gemmill, A.W. Towards Parenthood: An Antenatal Intervention to Reduce Depression, Anxiety and Parenting Difficulties. *Journal of Affective Disorders* **2011**, *130*, 385–394, doi:10.1016/j.jad.2010.10.045.
39. Mulcahy, R.; Reay, R.E.; Wilkinson, R.B.; Owen, C. A Randomised Control Trial for the Effectiveness of Group Interpersonal Psychotherapy for Postnatal Depression. *Arch Womens Ment Health* **2010**, *13*, 125–139, doi:10.1007/s00737-009-0101-6.
40. Ngai, F.; Wong, P.; Chung, K.; Chau, P.; Hui, P. Effect of Couple-based Cognitive Behavioural Intervention on Prevention of Postnatal Depression: Multisite Randomised Controlled Trial. *BJOG: Int J Obstet Gy* **2020**, *127*, 500–507, doi:10.1111/1471-0528.15862.
41. Oliveira-Ciabati, L.; Vieira, C.S.; Franzon, A.C.A.; Alves, D.; Zaratini, F.S.; Braga, G.C.; Sanchez, J.A.C.; Bonifácio, L.P.; Andrade, M.S.; Fernandes, M.; et al. PRENACEL – a MHealth Messaging System to Complement Antenatal Care: A Cluster Randomized Trial. *Reprod Health* **2017**, *14*, 146, doi:10.1186/s12978-017-0407-1.
42. Pezley, L.; Tussing-Humphreys, L.; Koenig, M.D.; Maki, P.; Odoms-Young, A.; Freels, S.; DiPiazza, B.; Cann, F.; Cares, K.; Depa, C.; et al. Feasibility of a Web-Based Intervention to Prevent Perinatal Depression and Promote Human Milk Feeding: Randomized Pilot Trial. *JMIR Form Res* **2022**, *6*, e32226, doi:10.2196/32226.
43. Pilkington, P.D.; Rominov, H.; Milne, L.C.; Giallo, R.; Whelan, T.A. *Partners to Parents:* Development of an Online Intervention for Enhancing Partner Support and Preventing Perinatal Depression and Anxiety. *Advances in Mental Health* **2017**, *15*, 42–57, doi:10.1080/18387357.2016.1173517.
44. Shorey, S.; Yang, Y.Y.; Dennis, C.-L. A Mobile Health App–Based Postnatal Educational Program (Home-but Not Alone): Descriptive Qualitative Study. *J Med Internet Res* **2018**, *20*, e119, doi:10.2196/jmir.9188.

45. Singla, D.R.; MacKinnon, D.P.; Fuhr, D.C.; Sikander, S.; Rahman, A.; Patel, V. Multiple Mediation Analysis of the Peer-Delivered Thinking Healthy Programme for Perinatal Depression: Findings from Two Parallel, Randomised Controlled Trials. *Br J Psychiatry* **2021**, *218*, 143–150, doi:10.1192/bjp.2019.184.
46. Jareethum, R.; Titapant, V.; Chantra, T.; Sommai, V.; Chuenwattana, P.; Jirawan, C. Satisfaction of Healthy Pregnant Women Receiving Short Message Service via Mobile Phone for Prenatal Support: A Randomized Controlled Trial. *J Med Assoc Thai* **2008**, *91*, 458–463.
47. Urizar, G.G.; Yim, I.S.; Rodriguez, A.; Schetter, C.D. The SMART Moms Program: A Randomized Trial of the Impact of Stress Management on Perceived Stress and Cortisol in Low-Income Pregnant Women. *Psychoneuroendocrinology* **2019**, *104*, 174–184, doi:10.1016/j.psyneuen.2019.02.022.
48. Zemestani, M.; Fazeli Nikoo, Z. Effectiveness of Mindfulness-Based Cognitive Therapy for Comorbid Depression and Anxiety in Pregnancy: A Randomized Controlled Trial. *Arch Womens Ment Health* **2020**, *23*, 207–214, doi:10.1007/s00737-019-00962-8.
49. Duffecy, J.; Grekin, R.; Hinkel, H.; Gallivan, N.; Nelson, G.; O'Hara, M.W. A Group-Based Online Intervention to Prevent Postpartum Depression (Sunnyside): Feasibility Randomized Controlled Trial. *JMIR Ment Health* **2019**, *6*, e10778, doi:10.2196/10778.
50. Haga, S.M.; Drozd, F.; Lisøy, C.; Wentzel-Larsen, T.; Slinning, K. Mamma Mia - A Randomized Controlled Trial of an Internet-Based Intervention for Perinatal Depression. *Psychol Med* **2019**, *49*, 1850–1858, doi:10.1017/S0033291718002544.
51. Kalmbach, David A.; Cheng, Philip; O'Brien, Louise M.; Swanson, Leslie M.; Sangha, Roopina; Sen, Srijan; Guille, Constance; Cuamatzi-Castelan, Andrea; Henry, Alasdair L.; Roth, Thomas; et al. A Randomized Controlled Trial of Digital Cognitive Behavioral Therapy for Insomnia in Pregnant Women. *Sleep Medicine* **2020**, *72*, 82–92, doi:10.1016/j.sleep.2020.03.016.
52. Loughnan, S.A.; Sie, A.; Hobbs, M.J.; Joubert, A.E.; Smith, J.; Haskelberg, H.; Mahoney, A.E.J.; Kladnitski, N.; Holt, C.J.; Milgrom, J.; et al. A Randomized Controlled Trial of "MUMentum Pregnancy": Internet-Delivered Cognitive Behavioral Therapy Program for Antenatal Anxiety and Depression. *J Affect Disord* **2019**, *243*, 381–390, doi:10.1016/j.jad.2018.09.057.
53. Matvienko-Sikar, K.; Dockray, S. Effects of a Novel Positive Psychological Intervention on Prenatal Stress and Well-Being: A Pilot Randomised Controlled Trial. *Women Birth* **2017**, *30*, e111–e118, doi:10.1016/j.wombi.2016.10.003.
54. Sun, Yaoyao; Li, Yanyan; Wang, Juan; Chen, Qingyi; Bazzano, Alessandra N; Cao, Fenglin Effectiveness of Smartphone-Based Mindfulness Training on Maternal Perinatal Depression: Randomized Controlled Trial. *Journal of Medical Internet Research* **2021**, *23*, doi:10.2196/23410.
55. Yang, M.; Jia, G.; Sun, S.; Ye, C.; Zhang, R.; Yu, X. Effects of an Online Mindfulness Intervention Focusing on Attention Monitoring and Acceptance in Pregnant Women: A Randomized Controlled Trial. *Journal of Midwifery & Women's Health* **2019**, *64*, 68–77, doi:10.1111/jmwh.12944.
